# The Place of Marginalization in Bioethics: Do We Need the Concept?

**DOI:** 10.1111/bioe.70103

**Published:** 2026-03-24

**Authors:** Elisabeth Langmann, Verina Wild

**Affiliations:** ^1^ Ethics of Medicine, Institute for Ethics and History of Health in Society, Faculty of Medicine University of Augsburg Augsburg Germany

**Keywords:** bioethics, concept, health, marginalization, vulnerability

## Abstract

Marginalization is a widely studied phenomenon and recognized as a critical topic in relation to health, shaping health inequities, access to resources, health outcomes, and policy decisions. However, despite its normative importance for health and justice, its conceptual role in bioethics remains unclear. This paper critically examines how marginalization is addressed in bioethics, analyzing its meaning, conceptual usage, and potential for further normative theorization. By exploring available definitions and how marginalization appears in bioethical literature, recurring patterns and conceptual gaps are identified. The analysis reveals two major findings. First, while definitions from disciplines beyond bioethics provide valuable insights, marginalization has not been normatively theorized as a distinct conceptual phenomenon within bioethics. Instead, dynamics of marginalization are largely addressed through established bioethical concepts, such as vulnerability or epistemic injustice. Second, it stands out that the contemporary redefinition of vulnerability in bioethics increasingly incorporates discussions on systemic exclusion and structural injustice, both central to marginalization. This raises the question of whether marginalization requires distinct conceptualization in bioethics or if its core dimensions are already encompassed by existing approaches and concepts. Consequently, this paper argues that further conceptual debate on marginalization is needed to assess its potentially distinct contributions to bioethics. It develops arguments for making space for the concept of marginalization within bioethics in its own right. To address these conceptual gaps, this paper calls for a more explicit normative analysis of marginalization to establish its role in bioethical discourse and to clarify its implications for equitable health, healthcare practices, and policies.

## 1 Introduction

Social inequities in health are a central topic of research and widely discussed across disciplines. In recent years, a wealth of studies has documented how systemic exclusion shapes access to care, opportunities, and resources, reinforcing disparities in health and health outcomes [1, 2, 3, 4]. Especially in the field of public health, dynamics of marginalization are increasingly recognized as key social determinants of health rather than consequences of inequities [5].

Social determinants of health, such as working conditions, healthcare accessibility, education, and discrimination, are known to critically shape inequities in access to health‐promoting resources [5, 6, 7, 8]. This includes policies that tie access to care to legal residency or employment [9], neglect certain diseases [10, 11], or enact public health strategies that fail to account for the diversity of needs [12, 13]. As such, marginalization is an active process that systematically influences who has access to resources and opportunities, who is prioritized in receiving care, whose voices are heard, and whose needs remain overlooked. Recent contributions, such as a review by Baah and colleagues, analyze marginalization within the framework of social determinants of health, emphasizing social position as a key link between systemic exclusion and health inequities [5].

For years, institutions such as the World Health Organization (WHO) have also emphasized the importance of addressing social determinants of health and their effect on so‐called “marginalized groups” [14, 15]. Similarly, national institutions, such as Germany's Robert Koch‐Institute (RKI), have pointed to social inequalities in their publications [16, 17]. However, while such initiatives address barriers to healthcare access for certain groups, they often focus on the need for inclusion into existing structures rather than addressing how these structures themselves contribute to marginalization.

Within bioethics, more and more attention is given to the topic of marginalization. Scholars like Cheema et al. argue that “[i]ncreasing the visibility of marginalized voices is fundamental to bioethics,” [18] a stance reflecting the field's development and evolving priorities. This growing focus is evident not only in the wide range of topics discussed, such as the impact of intersectional discrimination and various forms of injustice, but also in the application of specific lenses that bring these issues to the forefront. Concepts such as epistemic injustice, as well as approaches addressing intersectionality, have become central tools for examining how marginalization manifests in healthcare and society [19, 20, 21, 22, 23]. Rogers et al. capture this development noting a “rising generation of bioethicists interested in marginalization and discrimination, voice and power, and comfortable with using qualitative and experiential data to inform normative reflection.” [24]

Moreover, the strong attention to justice‐related issues within bioethics underscores the discipline's commitment to addressing systemic inequities. For example, feminist scholars have long emphasized the need to critique power dynamics, identify and amplify excluded voices, and analyze the ways in which structural and epistemic injustices shape health outcomes [18, 25, 26, 27]. Collectively, these contributions have supported the groundwork for deeper engagement with marginalization as a systemic and ethical concern within bioethics.

However, while marginalization is widely recognized as a critical issue, there is a surprising lack of normative and conceptual discussions within bioethics, in comparison to those that, for example, vulnerability has received. This raises key questions about how marginalization is understood in the field and whether further conceptual development is necessary. To address this gap, this article critically examines how marginalization is used and discussed within bioethics, exploring its meaning and its potential as an analytical lens or concept and considering what it could contribute to bioethical discourse. Specifically, the article reflects how marginalization is discussed in adjacent disciplines, its broader implications, and what this means for the field of bioethics. By engaging with existing definitions and uses, it seeks to lay the groundwork and open a conversation about the role of marginalization in bioethics and its significance for understanding, criticizing, and ultimately improving structural inequities in health and healthcare, while also examining whether marginalization requires conceptual attention in its own right within bioethics.

## 2 The Conceptual Significance of Marginalization in Bioethics

In many health‐related disciplines, such as public health and health sociology, marginalization as a dynamic of inequity is evident in an expanding body of research addressing social justice, discrimination, and othering in relation to health [28]. Socio‐empirical studies and broader philosophical contributions highlight dynamics of marginalization, drawing attention to specific groups being affected as well as to systemic patterns of exclusion shaping access to opportunities, resources, and healthcare as well as broader social determinants of health and health outcomes [29, 30, 31, 32, 33]. These discussions suggest that marginalization could or even should be a central aspect for understanding and addressing systemic inequities. To work towards a conceptual understanding within bioethics, we first reflect on existing definitions and uses across different disciplines. Looking across disciplines will help us to consider how existing definitions and uses are related to, or might inform, a deeper conceptual and normative engagement within bioethics. Accordingly, the following influential definitions from the fields of philosophy, nursing, psychology, and science and technology studies (STS) are explored:

Iris Marion Young, an influential American political theorist and socialist feminist, identified marginalization in her work *Justice and the Politics of Difference*, as one of the “five faces of oppression” [34]. For Young, marginalization is not an isolated event, but a structural and systemic process deeply embedded in societal structures and cultural norms and therefore in the fabric of society. According to her, marginalization systematically excludes certain groups from participating fully in different aspects of society, denying access to essential resources, opportunities for meaningful work, and social recognition, relegating them to positions of societal invisibility and disempowerment [34]. The description of marginalization by Young draws attention to its deeply entrenched and harmful consequences. In fact, she writes: “Marginalization is perhaps the most dangerous form of oppression. A whole category of people is expelled from useful participation in social life and thus potentially subjected to severe material deprivation and even extermination.” [34] Young thus suggests that marginalization not only entails exclusion but also the significant harm that results from being pushed to the margins of society.

Central to Young's analysis is the recognition that marginalization is dynamic rather than static. It appears to evolve in ways that sustain and reinforce existing power hierarchies through mechanisms such as discriminatory labor markets, restrictive housing policies, or inequitable access to healthcare [35]. As a variety of studies have shown, intersecting mechanisms such as these are known to deepen disadvantage, reinforce systemic inequalities, and reproduce marginalization in new and evolving forms [22, 36, 37, 38, 39, 40, 41]. Concerning these mechanisms, Young calls for moving beyond surface‐level solutions, advocating for a shift in focus from redistribution to systemic transformation of the societal structures and norms that perpetuate exclusion [34]. For public health and bioethics, this can be seen as a crucial insight as addressing marginalization may not only require understanding its structural roots but also imagining and working toward systems that foster inclusion and justice.

In nursing science, Hall and colleagues have contributed significantly to understanding marginalization as a guiding concept, defining it as “the process through which persons are peripheralized on the basis of their identities, associations, experiences, and environment.” [42], Their conceptual approach identifies properties such as power, voice, and liminality, later expanded to include a global perspective with elements like constraint, eurocentrism, and economics. As it is highlighted that the concept of marginalization remains work in progress and germane to nursing, the focus is mainly on how marginalization operates. By offering this theoretical perspective, Hall and colleagues aim to identify the diverse consequences of marginalization, emphasizing its various manifestations and the ways it operates across different contexts [43]. Later on, Hall and Carlson also provide a more comprehensive definition: “Marginalization is defined here as a process by which persons or groups are sociopolitically peripheralized from dominant, central experiences, that is, deprived of mobility, control over self will, and/or critical resources; indignified and humiliated; exposed to toxic environments; and/or exploited physically or mentally, such that they are at increased safety, health, social, and political risk.” [44] This expanded understanding reflects an effort to incorporate the structural, relational, and performative dynamics of marginalization, highlighting its role in sustaining harm and exclusion, and is therefore well suited to contribute to a more robust conceptualization of marginalization within bioethics.

More recently, Fluit et al., with affiliations in psychology, STS and social research, have conducted a comprehensive overview of how social marginalization has been understood and studied across disciplines [28]. Their scoping review, spanning 50 years and over 1100 papers, reveals both the breadth and fragmentation of marginalization as a concept. By identifying diverse key topics and the broad spectrum of groups impacted by marginalization, Fluit et al. illustrate its far‐reaching effects across contexts such as health, socio‐demographics, and discrimination. However, the review also emphasizes the fragmented use of the term, noting that its lack of a clear, consistent definition poses challenges for its application across disciplines. This issue is particularly relevant for bioethics, where conceptual clarity is essential for normative analysis. Additionally, the risk of vague or indiscriminate use of the term marginalization is pointed out, exhibiting the potential to reinforce reductive categorizations rather than addressing the structural mechanisms that sustain exclusion [28]. As such, the findings of the scoping review support that marginalization should not be interpreted as merely a static label or identity, but rather as a dynamic, relational process that is deeply embedded in systemic inequities. This aligns with Young's call to focus on systemic transformation [34] as well as Hall's emphasis on power, voice, and liminality [43, 44].

To address the conceptual fragmentation, Fluit et al. propose an integrated definition of marginalization as a “multifaceted concept that refers to a context‐dependent social process of “othering”—where certain individuals or groups are systematically excluded based on societal norms and values—and the resulting experience of disadvantage.” [28] This definition echoes the structural and relational dimensions highlighted by Young and Hall, while also emphasizing the role of societal norms in producing and perpetuating exclusion.

In contrast to the explicit definitions of marginalization offered by Young, Hall, and Fluit et al., analyzing bioethical literature concerning marginalization shows a variety of patterns. What stands out prominently is that marginalization is not defined explicitly or treated as a distinct concept. While some contributions reference Young's understanding of marginalization [26], this perspective is neither widely shared nor consistently and explicitly applied within the bioethics literature. At the same time, marginalization is widely recognized as a key dynamic of injustice. However, up to now, the focus has been on examining the empirical manifestations of marginalization. Mostly, it is understood as an experience and a consequence of broader social injustices. This raises questions about whether bioethics needs a distinct, normative conceptualization of marginalization at all.

Thus far, marginalization tends to be discussed in bioethics indirectly through other specific lenses and approaches such as vulnerability or epistemic injustice, which are established within bioethics [10, 26]. These concepts frequently appear in discussions of how certain groups *experience marginalization*, such as those affected by racism, ableism, sexism, or ageism, face exclusion or limited access to resources and opportunities [45, 46, 47]. Particularly in the context of epistemic injustice, relational aspects of marginalization emerge, including discussions of testimonial and hermeneutic injustice, which highlight how voices *are marginalized* and where systemic barriers hinder groups from understanding or interpreting experiences within healthcare systems [10, 11]. This shows further that marginalization is addressed in indirect, subordinated, fragmented and rather empirically accessible ways, lacking a clearly defined understanding or cohesive conceptual discussion in bioethics. Moreover, the reliance on other terms such as epistemic injustice and vulnerability as dominant lenses for understanding and discussing marginalization suggests that marginalization remains conceptually underdeveloped as a stand‐alone lens. For example, many discussions—especially those influenced by feminist philosophy and bioethics—draw on epistemic injustice to examine marginalization specifically in relation to knowledge and power [10, 11, 23, 48, 49, 50, 51]. Vulnerability, by contrast, is used more broadly to address systemic exclusion and inequities in the context of health [50, 52, 53, 54]. While critiques surrounding vulnerability rarely frame marginalization as their explicit focus, they nonetheless reveal dynamics of exclusion that are central to marginalization. This seems to be especially the case with the redefined understanding of vulnerability, by which it is no longer seen as an inherent characteristic of certain individuals or groups but rather as situational and shaped by broader structures [55, 56, 57]. This shift in how vulnerability is understood closely aligns with how marginalization is discussed in other disciplines, raising further important questions about whether marginalization requires distinct conceptualization in bioethics, or whether the evolving view on vulnerability already sufficiently encompasses its key dimensions.

While research has extensively documented dynamics of marginalization as processes of structural exclusion shaping health and healthcare, its status as a distinct normative concept within bioethics remains unclear. Importantly, this uncertainty does not concern the descriptive or empirical relevance of marginalization, which is well established across health and healthcare research, but rather its potentially added normative and analytical value as a distinct concept within bioethical inquiry. The question, therefore, is not whether marginalization matters descriptively, but whether treating it as a concept in its own right adds normative and analytical depth beyond existing approaches. Thus, in the next section, we turn to vulnerability as the topic most explicitly overlapping with and potentially absorbing the conceptual space of marginalization in bioethics. We examine the evolution of vulnerability as a concept, its role in addressing systemic exclusion, and its implications for a separate, deeper conceptual and normative engagement with marginalization in bioethics.

## 3 Vulnerability in Bioethics

Vulnerability has been a central concept in bioethics for decades and is widely recognized, discussed, and analyzed in relation to health, medical research, and clinical care. However, its meaning has shifted significantly over time, expanding beyond its initial focus on protecting specific groups from harm to a broader concern with structural and systemic disadvantage. This shift highlights both empirical and normative dimensions of vulnerability, describing real‐world mechanisms of exclusion, while also raising ethical concerns about justice, autonomy, and agency [58]. Overall, bioethics has broadened its focus on autonomy by increasingly emphasizing justice, agency, and structural transformation, a shift also reflected in discussions on vulnerability and related topics [26, 55, 58]. At the same time, the corresponding discussions are not only theoretical but describe systemic conditions that shape access, exclusion, and harm in concrete ways. Thus, while these terms highlight empirical realities of social inequality, their use in bioethics also carries normative weight, shaping how ethical concerns are framed and addressed.

To clarify how vulnerability has shaped bioethics, it is important to trace its historical trajectory, examining its introduction and how it has evolved (significantly) as a term and concept over time. Initially, vulnerability gained prominence through key ethical guidelines developed in response to historical maltreatment and abuses, including the human experimentation during World War II or the Tuskegee Syphilis Study [59, 60]. These prominent cases, among others, highlighted profound failures in protecting research participants, particularly those belonging to groups at heightened risk of harm and exploitation.

In reaction to this, the Nuremberg Code (1947) laid the foundation for research ethics, emphasizing voluntary consent and protections against coercion [61]. Building on these principles, the Declaration of Helsinki (1964) articulated ethical principles for medical research involving human participants [62]. However, early versions of the Declaration lacked explicit reference to vulnerability, focusing instead on protecting individuals from harm in narrowly defined categories, such as those individuals expected to be unable to give informed consent. This gap was later addressed in the Belmont Report (1979) [63], which articulated core principles of respect for persons, beneficence, and justice, highlighting the need to safeguard populations at heightened risk of harm or exploitation [64]. Together, these milestones established vulnerability as a critical concern in bioethics, laying the groundwork for its later expansion beyond research settings to clinical care and public health.

Over time, the concept of vulnerability broadened and was increasingly applied to specific groups perceived as requiring additional safeguards. Groups, such as older adults, children, prisoners, pregnant women, or individuals with disabilities, were often categorized as vulnerable due to presumed increased limitations in autonomy, consent capacity, or susceptibility to coercion [65]. While this approach has practical relevance, it has been subject to growing criticism for oversimplifying vulnerability and reinforcing static categorizations that risk perpetuating overprotection, victimization and exclusion [56, 66, 67]. Thus, it has been argued that the tendency toward categorizing groups as vulnerable promotes a harmful perception of homogeneity among the members of the respective group, overlooking intragroup differences, which consequently carries the risk of stereotyping and prejudice, while losing sight of specifically existing vulnerabilities [68, 69, 70]. Additionally, this approach has been criticized for potentially undermining autonomy, as it may imply that all individuals within a designated vulnerable group lack decision‐making capacity. This, in turn, can lead to paternalistic benevolence or, in the context of clinical research, discriminatory practices—either unjustly excluding certain populations from participation or disproportionately targeting them [70, 71, 72]. Further concerns arise from the broad application of the term, as labeling an increasing number of groups of people as vulnerable without clearly defining specific characteristics that justify their classification, there is a risk of rendering the concept overly broad, thereby potentially diminishing its practical utility in bioethics and public health [66].

Following up on these critiques, feminist bioethics has played a significant role in redefining the concept of vulnerability in medicine, medical research, and more recently also public health. Within this process, it has been argued to abandon the categorization of specific groups as vulnerable toward a more nuanced understanding of vulnerability as situational, layered, and dynamic [57, 69, 73]. This reconceptualization not only challenges static labels but, more broadly, also shifts the focus from protectionism towards issues of justice, empowerment, and agency [58]. In contrast to the categorization as vulnerable groups, the redefined understanding emphasizes that vulnerability is not merely an inherent aspect of human beings but is actively created and shaped within social, political, and economic contexts. Therefore, rather than focusing on who is vulnerable, feminist bioethics call for analyzing the mechanisms that create vulnerability, shifting the focus to *why* and *how* vulnerability arises [55].

This perspective has been widely applied to illustrate how structural conditions shape lived realities in ways that make certain individuals and groups more susceptible to harm [74, 75, 76, 77, 78, 79]. At the same time, feminist bioethics has critically examined the risks of categorizing groups as inherently vulnerable. As mentioned above, scholars argue that this framing reinforces stereotypes, oversimplifies diverse experiences, and overlooks structural determinants of exclusion [55, 57, 77, 80]. In ageing, for example, defining older adults as inherently vulnerable disregards economic policies, healthcare infrastructure, and other systemic factors shaping exclusion [67, 79, 81]. Rather than addressing these structural determinants, the broad application of vulnerability risks reducing ageing to a homogeneous, deficit‐based category, reinforcing paternalistic assumptions and limiting agency [67].

On a theoretical level, for instance, Luna's concept of “layered vulnerability” captures this complexity by demonstrating how multiple, intersecting factors—such as gender, race, socioeconomic status, or age—combine to shape and intensify layers of vulnerability. These intersecting layers reveal how systemic forces might work together to compound disadvantage, challenging us to move beyond individual circumstances and examine the role of systemic disempowerment, inequities, and social injustices in creating and perpetuating vulnerability. This can be observed, for example, considering a postmenopausal woman living with endometriosis, a condition often misunderstood and dismissed as a “disease of reproductive age.” [82, 83] Her vulnerability stems not primarily from her condition but from the intersecting structural and social biases embedded in healthcare systems. Ageist assumptions frequently lead to her symptoms being trivialized as “normal aging” or dismissed as inevitable menopausal changes [83, 84]. At the same time, the lack of research and clinical guidelines for managing endometriosis beyond the reproductive age produces vulnerabilities and results in healthcare providers lacking interpretative resources to offer appropriate care, leaving patients to navigate a system that overlooks needs and reinforces existing inequities, such as dismissal of symptoms and unequal access to care [10]. These layers of vulnerability, shaped by systemic ageism, gendered assumptions, and dynamics of injustice, are not inherent or static but can be reduced or eliminated with healthcare systems prioritizing research into older women's health, addressing biases in medical training, and developing evidence‐based guidelines that acknowledge the occurrence of endometriosis throughout the lifespan [10].

Thus, vulnerability has undergone extensive development and theorization within bioethics, in recent years particularly in feminist scholarship. Thereby, it has been redefined in ways that capture systemic exclusion [57]. This redefinition has increasingly positioned vulnerability not just as a category but as a heuristic tool—a lens for identifying systemic disadvantage and conditions of moral harm, rather than labeling predefined groups as inherently vulnerable [70]. This evolving perspective recognizes that vulnerability can change over time, with sources and/or layers emerging, intensifying, or vanishing in response to shifting circumstances [57].

Vulnerability is thus increasingly understood as relational and context‐dependent, emphasizing how social, political, institutional, and economic conditions create, sustain and have the power to reduce exclusion [56, 57, 69, 73]. By accentuating such nature of vulnerability, bioethics has expanded its analytical scope to better include the intersecting roles of different contexts – and with that, the potential to highlight dynamics of marginalization. For instance, it is pointed out how systemic inequities, such as inadequate pensions, gender disparities, and poor infrastructure, increase vulnerabilities among older adults and simultaneously reinforce their marginalization [67, 79, 81]. From this perspective, vulnerability serves as a lens for identifying structures of harm, while marginalization remains a distinct, empirically traceable, process shaped by systemic exclusion.

Nonetheless, the redefined understanding of vulnerability has increasingly shifted toward (also) capturing structural conditions of exclusion and injustice, which raises the question of how it relates to marginalization, and whether it absorbs the need for a distinct conceptual engagement with marginalization. As discussed above, marginalization has not been established as a concept in its own right in bioethics. Therefore, the following discussion engages with the question whether such a distinct and explicit conceptual and normative theorization is required, examining potential benefits and limitations thereof.

## 4 Marginalization in Bioethics: Do We Need a Concept?

Vulnerability and marginalization are both widely discussed topics, each playing a critical role in bioethics and health‐related disciplines. Whereas vulnerability is a long‐established and extensively discussed concept within bioethics, marginalization has surprisingly not undergone a similar process. It remains unclear whether engaging further with marginalization as a concept would offer distinct contributions beyond those already captured by vulnerability, or whether it risks reiterating existing discussions. Furthermore, it invites reflection on whether vulnerability is sufficient to detect and address marginalization in the context of health, or if there is a need for a conceptual and normative theorization of marginalization.

Drawing a comparison between vulnerability and marginalization is challenging, as the two have distinct origins and have been taken up differently within bioethics. Therefore, taking into account the linguistic roots of the terms “vulnerability” and “marginalization” can be instructive. The term marginalization, derived from the Latin word *margo*, meaning edge or border, refers to the act of positioning or pushing individuals or groups away from societal, economic, and political centers. In comparison, vulnerability derives from the Latin word *vulnus*, meaning wound and conveys a potential and an openness to harm, emphasizing susceptibility and exposure.

Within bioethics, vulnerability has evolved to highlight the risks and dependencies that individuals or groups face in specific contexts. Its linguistic roots carry a connotation of passivity, framing those labeled as vulnerable to be inherently at risk. At the same time, vulnerability has undergone extensive theoretical and normative development, making it a powerful tool for identifying and addressing systemic inequities, power imbalances, and the dynamics of marginalization. This transformation, particularly shaped by feminist bioethics, has deepened its usefulness for recognizing and addressing structural and epistemic injustices. As a result, we can conclude that the former, rather passive connotation of vulnerability, mainly used in relation to consent in biomedical research, had little overlap with the discussions of marginalization. However, the redefinition of vulnerability towards a more active, process and structure‐oriented concept, sensitive also to dimensions of justice and exclusion, has increasingly blurred the contrast to the way marginalization is used and discussed in adjacent disciplines.

Building on these considerations, it is uncertain how a stand‐alone conceptualization of marginalization in bioethics might differ from existing analyses. At the same time, the absence of a distinct concept could offer greater adaptability in addressing its complexities, as marginalization is a heterogeneous phenomenon that manifests in diverse ways across contexts. A more rigid conceptualization could risk oversimplifying the dynamic and context‐dependent nature of marginalization, making it difficult to account for the shifting and intersectional ways in which exclusion operates. Instead, bioethics might also benefit from addressing marginalization through established lenses, such as vulnerability or epistemic injustice, that allow for greater contextual flexibility.

Beyond these conceptual considerations, a further challenge in introducing marginalization into bioethics “in its own right” can be associated with the term itself. It inherently implies pushing or being pushed to the margins [28, 34, 44]. While vulnerability is understood as *conditio humana* and therefore (at least partly) as a universal characteristic, marginalization remains linguistically tied to exclusion, harm, and deprivation. Framing a group as marginalized may, even more than the language of “vulnerability,” risk reinforcing stigmatizing narratives by focusing predominantly on what the group lacks or experiences as harm, and thereby firmly establishing the privileges of those at the center, rather than addressing the systems and mechanisms responsible for exclusion [28]. This ties into broader critiques of the so‐called deficit discourse, which suggest that emphasizing disadvantage can end up essentializing groups affected by marginalization and shifting attention away from the structural forces that drive exclusion and injustice [85, 86]. This concern parallels critiques of the “vulnerable groups” discourse in bioethics described above, where labeling groups as vulnerable has often led to oversimplifications or stereotyping.

Thus, it remains questionable if bioethics should strengthen another term that has the potential of framing groups primarily through a deficit‐oriented lens, focusing on what has been denied to the affected group rather than focusing on the structures and mechanisms responsible for that exclusion. This could be interpreted as limiting for the practical utility of a concept of marginalization in bioethics, and potentially also for contemporary uses of vulnerability.

However, despite these challenges, the theoretical conceptualization of marginalization might also offer distinct advantages for bioethics. First, the absence of a differentiated and nuanced conceptualization of marginalization within bioethics might have left critical gaps in how we understand and address systemic exclusion. Examples of this can be found within bioethics and beyond, where the categorization of “vulnerable groups” remains widely used despite ongoing criticism [67, 87, 88, 89]. While the role of systemic biases and structural inequalities in producing and sustaining a vulnerable position is in some cases acknowledged, it is also often overlooked or insufficiently addressed. An example of this is seen in research and policies concerning refugees, where vulnerability is often framed as an inherent consequence of displacement, rather than a result of systemic barriers [90, 91, 92].

This need for a more explicit focus on systemic mechanisms of exclusion, oppression and othering is echoed by recent scholarship calling for bioethics to take a more active role in advancing justice. For instance, Fletcher et al. argue that bioethics has historically failed to engage with the institutionalized biases that shape research priorities, policy development, and healthcare access, thereby reinforcing systemic inequities [93]. Similarly, Fabi and Goldberg critique bioethics' funding priorities, demonstrating how the field has focused disproportionately on biomedical and technological advancements while failing to sufficiently engage with structural determinants of health [94]. Their work highlights how funding gaps perpetuate epistemic and social injustice by neglecting research that could address inequities rooted in systemic marginalization [94]. Furthermore, Newman emphasizes the need to decenter dominant analytical lenses in bioethics and refocus on institutional change, arguing that the field must move beyond simply increasing representation toward addressing the deeper structural mechanisms that perpetuate exclusion [25]. Chung further reinforces this critique by highlighting how structural vulnerability and epistemic injustice intersect to sustain inequities within healthcare systems [50]. Taken together, these perspectives suggest that bioethics needs to move beyond acknowledging inequities and actively engage with the systemic processes that sustain marginalization.

Given these critiques, the normative theorization and conceptualization of marginalization may have the potential to shift the focus more toward systemic exclusion and oppression as well as the active processes that promote and sustain marginalization. By centering exclusionary and oppressive mechanisms, a concept of marginalization might offer a more explicit engagement with the structural roots of injustice, while avoiding the static or stigmatizing connotations often associated with traditional uses of vulnerability. In this sense, a concept of marginalization appears beneficial for bioethics and health‐related disciplines, providing an analytical lens that centers processes of marginalization themselves, rather than subsuming them under broader discussions of risk of harm, inequity or discrimination.

Thus, conceptualizing marginalization could offer a valuable perspective for exploring justice in bioethics in a more nuanced and structural way, particularly by emphasizing accountability for dismantling entrenched mechanisms of exclusion and oppression. This lens can encourage a deeper focus on the policies, cultural narratives, and institutional practices that sustain exclusionary and oppressive dynamics. Importantly, this approach could involve amplifying the voices of those who experience marginalization while critically examining the complex and structural mechanisms that sustain it. Centering these perspectives could, in turn, support shifting the focus toward inclusion and systemic changes necessary to address persistent inequalities.

A distinct conceptualization of marginalization could align with this by equipping bioethics with analytical tools to explicitly address exclusionary and oppressive mechanisms, rather than relying on less specific concepts such as vulnerability to capture these systemic injustices indirectly. It might also offer new approaches to address issues of access, recognition, and belonging, which are deeply shaped by societal power dynamics. By foregrounding these exclusionary processes, marginalization as a lens for justice would resonate with bioethics' growing emphasis on structural injustices and its potential for transformative change. As such, we suggest that developing marginalization as a concept within bioethics can open up new possibilities to address critical gaps left out by existing approaches, offering a more explicit way to engage with systemic inequities.

## 5 Conclusion

This paper critically examines the place of marginalization within bioethics, questioning whether its explicit conceptualization and normative theorization can provide distinct analytical value beyond existing approaches such as vulnerability. The analysis shows that while concepts like vulnerability have undergone significant theorization, marginalization—though widely discussed in relation to systemic health inequities—remains vague and under‐theorized within bioethics. Instead, bioethical discussions often approach marginalization through the redefined lens of vulnerability, which has increasingly incorporated structural and relational dimensions. This gap suggests a missed opportunity to explicitly engage with the active processes and systemic mechanisms that sustain social exclusion, oppression, harm, and deprivation.

By examining existing definitions of marginalization and their implications, this paper explores the potential benefits of developing marginalization as a stand‐alone concept in bioethics. While questions remain, it is suggested that such a concept can offer a complementary and distinctive lens for addressing justice and systemic accountability in the context of health. Based on this analysis, we argue that developing marginalization as a distinct concept can provide an important lens to foreground exclusionary and oppressive mechanisms and advocate for transformative change in bioethics. As such, this work invites further exploration and normative theorization of marginalization, aiming to fill critical gaps left by existing approaches within bioethics and contribute to the pursuit of justice in health‐related disciplines.

## Ethics Statement

The authors have nothing to report.

## Conflicts of Interest

The authors declare no conflicts of interest.
